# Lipomas are associated with a higher prevalence of metabolic syndrome components: a multicenter cross-sectional study

**DOI:** 10.3389/fendo.2025.1721570

**Published:** 2025-12-02

**Authors:** Ori Berger, Shaked Menashe, Shiri Damti Geva, Reychel Yakubov, Maor Ben Yehuda, Mor Peleg, Ran Talisman

**Affiliations:** 1 Department of Plastic Surgery, Barzilai Medical Center, Ashkelon, Israel; 2 Department of Plastic Surgery, Shamir Medical Center, Rishon LeZion, Israel; 3 Department of Otolaryngology and Head and Neck Surgery, Galilee Medical Center, Nahariya, Israel; 4 Department of Information Systems, University of Haifa, Haifa, Israel

**Keywords:** lipoma, metabolic syndrome, obesity, epidemiology, diabetes mellitus, dyslipidemia, adipose tissue dysfunction

## Abstract

**Introduction:**

Lipomas are the most common benign adipocytic tumors and are traditionally regarded as incidental findings with cosmetic significance. However, their frequent occurrence in adults with obesity and metabolic risk factors raises the possibility that lipomas may reflect systemic metabolic dysfunction rather than isolated adipose overgrowth. The present study evaluated whether adults with lipomas have a higher prevalence of metabolic syndrome components, obesity, dyslipidemia, hypertension, and type 2 diabetes mellitus (T2DM), compared with population benchmarks.

**Methods:**

We conducted a retrospective, multicenter, cross-sectional analysis of electronic health records from three Israeli hospitals (Barzilai, Shamir, and Galil Medical Centers) between January 2000 and December 2022. Adults aged ≥21 years with a clinical diagnosis of lipoma (ICD-9-CM 214) were included. Data were harmonized using the Observational Medical Outcomes Partnership (OMOP) Common Data Model and analyzed via the Lynx real-world health data platform. Prevalence of obesity, dyslipidemia, hypertension, and T2DM was compared against age- and sex-specific benchmarks from the 2023 Israeli Knowledge, Attitudes, and Practices (KAP) survey. Subgroup comparisons used Z-tests or exact binomial tests with α = 0.05, reporting absolute differences with 95% confidence intervals.

**Results:**

A total of 7,868 adults with lipomas were analyzed (mean [SD] age, 53.0 [15.0] years; 53.6% women). Compared with population benchmarks, lipoma patients showed consistently higher prevalence of all four metabolic traits. Dyslipidemia was most overrepresented, followed by hypertension and obesity, while T2DM showed a uniform excess across all age and sex subgroups. Clustering of three or more metabolic traits—consistent with metabolic syndrome, was common after age 35 and most pronounced in midlife.

**Conclusion:**

Adults with lipomas exhibit a substantially higher burden of metabolic syndrome components compared with population norms. These findings suggest that lipomas may serve as visible clinical indicators of systemic metabolic dysfunction. Recognizing lipomas as potential cutaneous markers of cardiometabolic risk could improve early identification of individuals at risk for obesity-related and endocrine diseases and support integration of dermatologic and metabolic screening practices.

## Highlights

This multicenter, real-world study identifies lipomas as common but under-recognized cutaneous indicators of systemic metabolic dysfunction. By demonstrating a graded association between lipomas and metabolic clustering, our findings highlight lipomas as potential clinical markers for early detection of cardiometabolic disease. Recognition of these links may inform targeted screening and preventive care while providing insights into the shared biological pathways that connect adipose dysfunction, benign tumorigenesis, and possibly tumour immunity.

## Introduction

Lipoma, the most common benign tumor in humans, affects approximately 1%–2% of adults and is frequently encountered in dermatology, plastic surgery, and primary care practice. These lesions typically present as soft, mobile subcutaneous nodules composed of mature adipocytes and are histologically similar to normal adipose tissue (1, 2). Most lipomas follow an indolent course, and treatment usually consists of surgical excision. Although generally regarded as incidental findings with primarily cosmetic significance (3), lipomas occur more often in individuals with obesity between 40 and 70 years of age, may appear at multiple sites in 5%–15% of cases, and rarely undergo malignant transformation into liposarcoma (1).

Metabolic syndrome (MetS) is defined by the co-occurrence of abdominal obesity, dyslipidemia, hypertension, and hyperglycemia, and markedly increases the risk of cardiovascular disease, stroke, and several cancers (4). Its global prevalence continues to rise in parallel with the growing burden of obesity and type 2 diabetes mellitus (T2DM). Despite these trends, the potential relationship between lipomas and systemic metabolic dysregulation has not been rigorously evaluated at a population level. To date, no large, population-level study has systematically quantified the association between lipomas and metabolic traits.

The concept that lipomas may reflect systemic metabolic abnormalities rather than representing isolated tumors has long intrigued clinicians (5). Case reports have described lipomas in patients with diabetes (1, 2) or hyperlipidemia (1), and mechanistic studies have implicated adipocyte proliferation and lipid-metabolism pathways. Furthermore, mutations in the mitochondrial fusion gene MFN2 have been linked both to multiple symmetric lipomatosis and to features of MetS (6, 7), suggesting a potential biological bridge between localized lipoma formation and systemic metabolic disease. Collectively, these observations raise the hypothesis that lipomas may serve as visible markers of underlying metabolic dysfunction.

To address this question, we conducted a large multicenter, real-world study across three Israeli medical centers to evaluate whether adults with lipomas exhibit a higher prevalence of obesity, T2DM, dyslipidemia, and hypertension compared with population benchmarks. Our primary objective was to determine whether adults with lipomas carry a higher prevalence of metabolic syndrome components relative to the general population. We hypothesized that patients with lipomas would show an excess burden of obesity, dyslipidemia, hypertension, and T2DM, consistent with lipomas serving as clinical indicators of systemic metabolic risk.

## Materials and methods

### Study design and setting

We conducted a retrospective, multicenter, cross-sectional epidemiological study using electronic health records from three Israeli hospitals: Barzilai Medical Center, Shamir Medical Center, and Galil Medical Center.

Data were accessed through the Lynx platform in collaboration with the Kinneret Israel Health Data Lake, which harmonizes real-world healthcare data using the Observational Medical Outcomes Partnership (OMOP) Common Data Model. The Lynx platform has undergone independent quality validation for completeness and consistency of diagnostic, medication, and laboratory data.

All data were de-identified prior to analysis, and informed consent was not required. The study was approved by the Institutional Review Boards (IRBs) of all participating centers (Barzilai IRB approval number BRZ-0066-24). All procedures conformed to institutional and national regulations.

Data spanned January 2000 through December 2022, and analyses were conducted between March and August 2025.

### Study population and comparison group

Adults aged ≥21 years with a diagnosis of lipoma (ICD-9-CM 214) were identified from hospital inpatient and outpatient records. Because case identification was limited to hospital-based encounters, lipomas managed exclusively in community settings may not have been captured. The index date was defined as the earliest recorded lipoma diagnosis. Pathology confirmation was not required because case identification relied on diagnostic coding.

Exclusion criteria were limited to age <21 years or missing essential demographic data. Implausible laboratory or measurement values were removed for the affected trait but did not lead to exclusion from the overall cohort. Individuals without sufficient diagnostic, prescription, or measurement data for a specific metabolic trait were excluded from that trait’s analysis to ensure classification accuracy.

To identify as many at-risk patients as possible, we prioritized sensitivity over specificity, accepting a higher false-positive rate to minimize under-ascertainment of metabolic conditions. We did not perform imputation for missing data; records without timestamps or essential measurements were treated as out of scope rather than backfilled.

Because there is no reliable dataset of individuals confirmed to be lipoma-free, diagnosing lipomas typically requires imaging or full-body physical examinations that are not performed routinely, we could not assemble a true internal control group. Instead, we used age- and sex-specific prevalence estimates from the 2023 Israeli Knowledge, Attitudes, and Practices (KAP) survey (N = 5,808). This survey reports the prevalence of overweight and obesity (BMI ≥25 and ≥30 kg/m²) by age and sex but not continuous BMI values; thus, comparisons were limited to categorical prevalence.

Because all eligible hospital cases were included, no *a priori* sample size calculation was performed. Given the large sample size, the analysis had >90% power to detect a 5% prevalence difference compared with benchmarks at α = 0.05.

### Definitions of metabolic traits

Four traits were evaluated: obesity, type 2 diabetes mellitus (T2DM), dyslipidemia, and hypertension.

**Figure 1:**
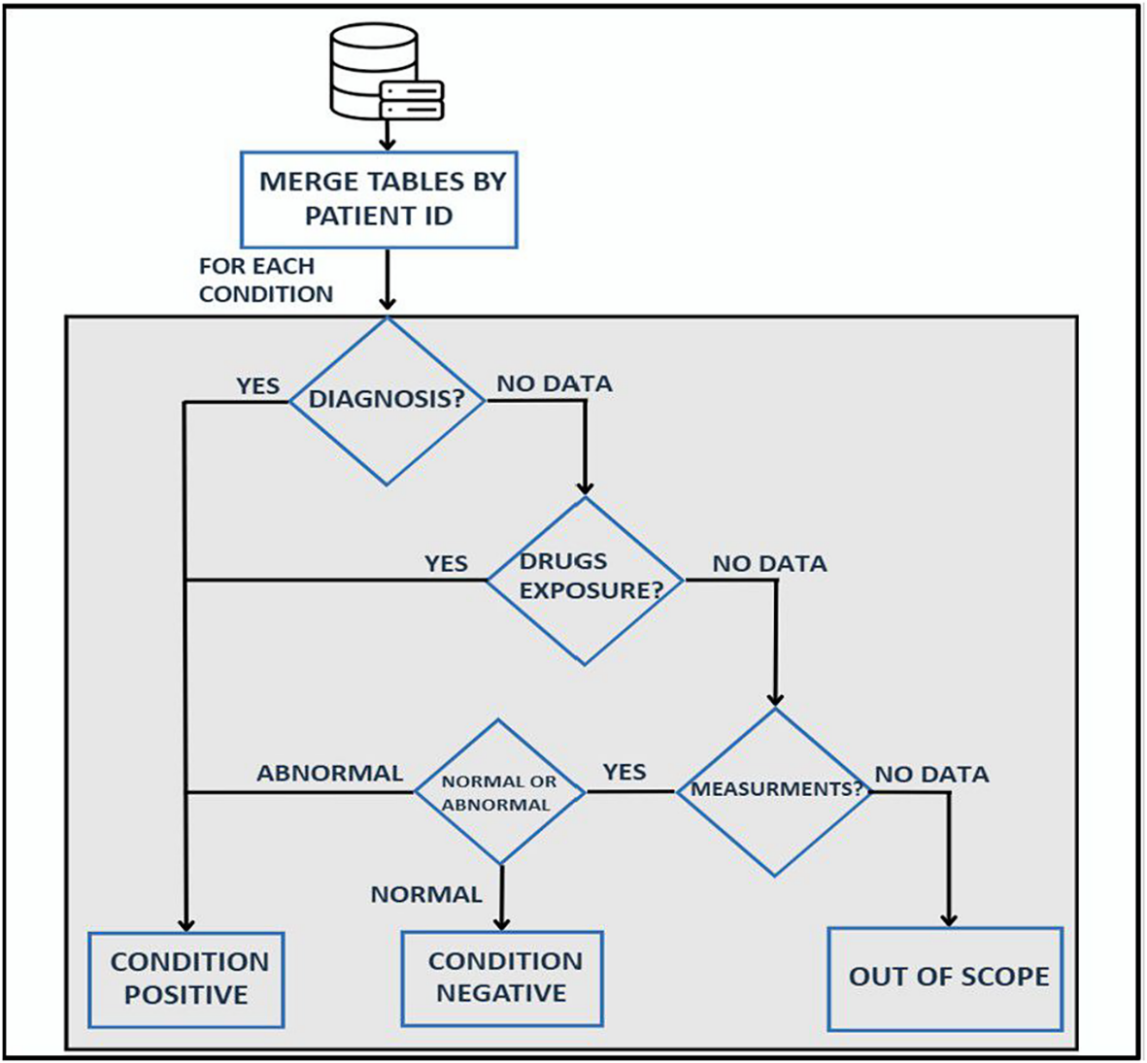
Hierarchical algorithm for metabolic trait classification. Flowchart of the rules used to classify obesity, type 2 diabetes mellitus (T2DM), dyslipidemia, and hypertension. For each trait, patients were considered positive if any of the following were present: a relevant diagnosis code, active medication exposure, or an abnormal measurement (laboratory or vital sign). Records with no data for a given trait were labeled out of scope for that trait. Abbreviations: T2DM, type 2 diabetes mellitus.

Each trait was classified as present if supported by diagnostic codes, chronic medication use, or abnormal laboratory values; absent if normal results were documented without diagnosis or medication; or excluded if no relevant data were available. Traits were assessed sequentially—diagnoses, then medications, then measurements. Implausible values were excluded, and no imputation was performed. Records lacking timestamps or essential measurements were considered out of scope (**Figure 1**, **Appendix S1**).

### Statistical analysis

Demographic and clinical characteristics were summarized using descriptive statistics. Categorical variables were reported as counts and percentages, and continuous variables as means (standard deviation [SD]) or medians (interquartile range [IQR]) depending on distribution.

BMI was analyzed categorically (≥25 and ≥30 kg/m²) to match benchmark definitions. Age was grouped in five-year bands to ensure comparability across subgroups. Laboratory and vital-sign variables (glucose, HbA1c, lipid levels, blood pressure) were analyzed both as continuous measures and dichotomized at clinical thresholds.

The primary objective was to compare the prevalence of obesity, T2DM, dyslipidemia, and hypertension in adults with lipoma against age- and sex-specific benchmarks from the 2023 KAP survey.

Secondary objectives included: Sex- and age-stratified analyses, Exploratory clustering of ≥3 metabolic traits (MetS-like composites), and Sensitivity analyses across hospital sites.

For each age-sex subgroup, the observed prevalence in the lipoma cohort (p̂) was compared with the benchmark (p_0_). Z-tests for proportions were applied when subgroup size was ≥30 and normal approximation assumptions were met (n · p_0_ ≥ 5 and n · [1 − p_0_] ≥ 5). If assumptions were not met, exact binomial tests were used. Subgroups with zero observed cases were excluded as no test statistic could be computed. Exact one-sided binomial tests were applied for small subgroups when at least one case was present but normality was violated.

Results are expressed as absolute differences with 95% confidence intervals (CIs) and relative risks (RRs) where appropriate. All tests were two-sided with α = 0.05. Because of multiple comparisons, P-values are interpreted as exploratory.

Analyses were performed in Python version 3.11 (Python Software Foundation, Wilmington, DE, USA) using the statsmodels (v0.14) and SciPy (v1.11) libraries.

Potential sources of bias included the lack of a confirmed lipoma-free control group, small or empty age-sex subgroups, and possible misclassification due to diagnostic coding, non-specific medication use, or transient laboratory abnormalities. To mitigate these issues, we used a hierarchical evidence framework, excluded physiologically implausible values, harmonized data via the OMOP Common Data Model, and performed sensitivity analyses across hospitals. Results were consistent across sites.

Sex (male/female), as recorded in the electronic health record, was included in all stratified analyses; gender identity was unavailable.

Exploratory analyses assessed clustering of ≥3 traits as “MetS-like composites”, compared with benchmarks using identical statistical methods.

Multivariable logistic regression was considered but not performed because person-level covariates (e.g., BMI, medications, laboratory and vital measurements) were unavailable for the external benchmark (KAP) dataset; therefore, age- and sex-stratified prevalence contrasts were used as the only analytically aligned approach across datasets. As a cross-sectional analysis of routinely collected records, there was no follow-up or attrition.

## Results

We identified 8,094 patients with a recorded diagnosis of lipoma across three hospitals (Barzilai, n=1,900; Shamir, n=4,194; Galil, n=2,000). After excluding 198 patients younger than 21 years and 28 patients with missing values, the final analytic cohort included 7,868 adults (Barzilai, n=1,857; Shamir, n=4,074; Galil, n=1,937).

Because this was a registry-based study using routinely collected health records, no patient attrition occurred. All included participants contributed data to at least one metabolic trait analysis, although availability varied by trait. A two-sample comparison showed no significant differences in trait prevalence across hospitals, justifying pooled analysis.

The analytic cohort included 7,868 adults with lipoma (mean [SD] age, 53.0 [15.0] years; median, 53.1 years; 53.6% women). Most patients were aged 45–64 years. Age distribution was 12.6% aged 21–34, 18.7% aged 35–44, 23.3% aged 45–54, 21.6% aged 55–64, and 23.9% aged ≥65 years. Sex distribution varied modestly by hospital but showed a slight female predominance overall (53.6%).

Data completeness for metabolic traits is summarized in **Table 1**. Among available measurements, the mean (SD) BMI was 28.05 (5.41) kg/m² (median, 27.4); 28.4% of measured individuals were classified as obese (BMI ≥ 30 kg/m²), representing 7.2% of the total cohort. Mean (SD) systolic and diastolic blood pressures were 131.2 (19.5) mm Hg and 75.8 (11.7) mm Hg, respectively. Mean (SD) HDL cholesterol was 41.6 (13.0) mg/dL, triglycerides 131.7 (74.6) mg/dL, glucose 145.7 (55.2) mg/dL, and HbA1c 7.2 (1.8%).

**Table 1:** Availability and missingness of metabolic variables in the lipoma cohort.

Variable	Available (%)	Missing (%)
Body Mass Index	2,253 (25.6)	5,615 (74.4)
Lipid Profile	1,007 (12.8)	6,238 (87.2)
HbA1c	1,630 (20.8)	6,861 (79.2)
Blood Pressure	1,479 (18.8)	6,389 (81.2)

Compared with benchmarks from the 2023 Israeli Knowledge, Attitudes, and Practices (KAP) survey, lipoma patients were older and more frequently obese (**Table 2**). Because the KAP survey reports only categorical prevalence of overweight and obesity by age and sex, comparisons were limited to those categories.

**Table 2:** Characteristics of adults with lipomas compared with the National Health Survey population.

Characteristic	National health survey (KAP, n=4,135)	Lipoma cohort (n=7,868)
Age, mean (SD), y	51	53.1
Women %	53.4	61.0
Men %	46.6	39.0
Obesity (BMI ≥30) %	17.7	25.4
Type 2 diabetes mellitus %	9.0	38.4
Hypertension %	17.9	64.1
Dyslipidemia %	23.8	83.8

Baseline demographic and clinical characteristics of adults with clinically diagnosed lipomas compared with participants in the Israeli national health survey.

BMI, body mass index; SD, standard deviation.

### Prevalence of metabolic traits

**Figure 2:**
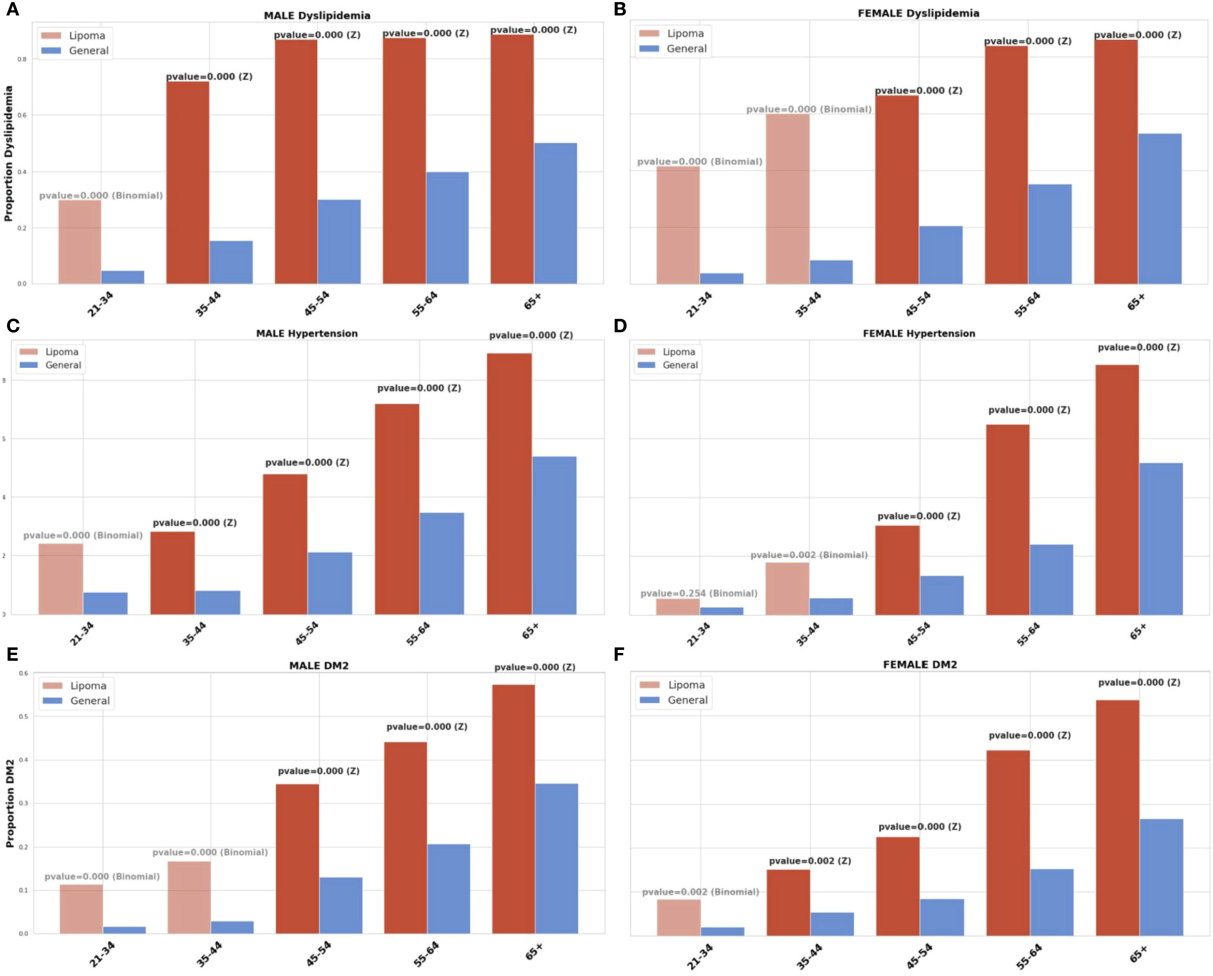
Prevalence of metabolic traits in adults with lipomas vs. population benchmarks. Age- and sex-stratified prevalence of **(a, b)** dyslipidemia, **(c, d)** hypertension, and **(e, f)** type 2 diabetes mellitus (T2DM) in the lipoma cohort (red) compared with the 2023 Israeli Knowledge, Attitudes and Practices (KAP) survey (blue). Error bars indicate 95% confidence intervals for cohort prevalence. Group differences were tested using two-proportion Z-tests, with exact binomial tests applied when cell counts were small; significance was set at *p* < 0.05. Abbreviations: CI, confidence interval; KAP, Knowledge, Attitudes and Practices; T2DM, type 2 diabetes mellitus.

**Figure 3:**
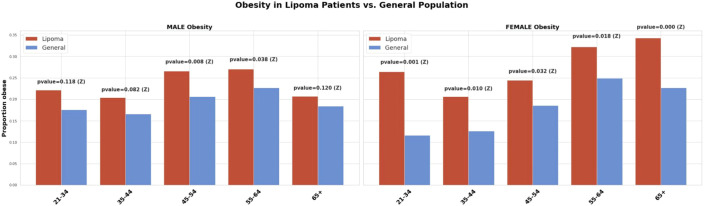
Prevalence of obesity in adults with lipomas vs. population benchmarks. Proportion with obesity (BMI ≥30 kg/m²) in the lipoma cohort (red) vs. KAP 2023 benchmarks (blue), stratified by sex and age group. Error bars show 95% CIs. Between-group contrasts used Z-tests (exact binomial tests when sparse); *p* < 0.05 was considered significant. Abbreviations: BMI, body mass index; CI, confidence interval; KAP, Knowledge, Attitudes and Practices.

The prevalence of dyslipidemia, hypertension, obesity, and type 2 diabetes was higher among patients with lipoma than among age- and sex-matched population benchmarks (**Figures 2**, **3**).

Dyslipidemia was significantly more prevalent among lipoma patients compared with the general population across all age and sex groups (p < 0.001 for all comparisons). Among men, prevalence rose from 30.0% in ages 21–34 years to 88.7% in those aged ≥65 years, versus 4.7% to 50.1% in the general population, respectively. Among women, prevalence increased from 41.7% at ages 21–34 to 86.2% at ≥65 years, compared with 3.9% to 53.1% in benchmarks. Both Z-tests and exact binomial tests confirmed statistical significance in every age stratum, with lipoma patients exhibiting dyslipidemia rates approximately 1.6- to 10.7-fold higher than population norms. The strongest relative excesses were observed in younger adults, suggesting that metabolic abnormalities are overrepresented even at early ages among lipoma patients.

Hypertension was also markedly overrepresented in lipoma patients. Among men, prevalence increased from 24.1% in ages 21–34 to 89.3% at ≥65 years, compared with 7.5% to 54.0% in the general population. Among women, rates rose from 5.6% to 85.4%, versus 2.7% to 51.8% in benchmarks. Most comparisons were significant at p < 0.001 by either Z-test or binomial test. The absolute differences ranged from ~3–6% in older adults to >15% in younger strata. As with dyslipidemia, the prevalence gap was largest in early adulthood, indicating that vascular comorbidities tend to appear earlier and more frequently in the lipoma cohort.

Type 2 diabetes was substantially more common in the lipoma cohort than in the general population across all ages (p < 0.001). Among men, prevalence rose from 11.3% in ages 21–34 to 57.3% at ≥65 years, compared with 2.0% to 34.6% in benchmarks. In women, the corresponding rates increased from 7.3% to 53.7% versus 2.1% to 34.6%. Both Z- and binomial tests indicated significant excesses in nearly every stratum. Relative differences were greatest in younger adults, exceeding 3-fold in those under 45 years, underscoring an early metabolic predisposition among individuals with lipoma.

Obesity showed smaller but consistent excesses among lipoma patients, particularly among women. In men, prevalence ranged from 20–27% across age groups compared with 17–23% in the general population, reaching statistical significance mainly in mid-life (p = 0.008–0.04). Among women, obesity prevalence rose from 26.4% at ages 21–34 to 34.3% at ≥65, compared with 11.6% to 22.7% in benchmarks (all p ≤ 0.03). The relative risk elevation was most pronounced in young and middle-aged women (approximately 2-fold), supporting a moderate but meaningful association between lipoma and adiposity.

### Clustering of metabolic traits

Across all three composite phenotypes—dyslipidemia+diabetes+hypertension (DDH), dyslipidemia+diabetes+obesity (DBD), and diabetes+obesity+hypertension (DBH), clustered metabolic abnormalities were consistently and substantially more common in lipoma patients than in benchmarks, with nearly all mid- and late-adult strata significant (mostly p<0.001). In men, DDH reached 46.6–57.1% at ages 55–64/≥65 versus 9.1–16.7% in the general population; DBD rose from 6.2% (35–44) to 17.5% (≥65) versus 0.4–4.2%; and DBH increased from 2.0% (35–44) to 14.1% (≥65) versus 0.4–6.3%. In women, DDH was 47.2% at ≥65 versus 14.5%; DBD was 16.8% at ≥65 versus 6.7%; and DBH was 21.0% at ≥65 versus 7.3% (all p ≤ 0.05). Taken together, these patterns indicate a dose–response: as more metabolic traits co-occur, the excess prevalence in the lipoma cohort widens, strengthening the association between lipoma and cardiometabolic clustering beyond that observed for individual traits.

## Discussion

In this multicenter, real-world study, adults with clinically diagnosed lipoma exhibited a significantly higher prevalence of metabolic syndrome and its core components. The excess strengthened with metabolic clustering. Clinically, these findings support using a lipoma encounter as a cue for a brief cardiometabolic screen (blood pressure, BMI or waist circumference, lipid profile, and fasting glucose or HbA1c), particularly in adults ≥35 years or when multiple lesions or other metabolic features are present.

Previous studies have long suggested that lipomas develop more frequently in individuals with metabolic risk factors. Epidemiologic data indicate that subcutaneous lipomas are most common in middle-aged adults, with multiplicity in 5–15% of cases (1). Case reports and small series have reported frequent co-occurrence with obesity, diabetes, dyslipidemia, and hypertension (8). More recent studies reinforce this pattern: spinal epidural lipomatosis has been identified as a manifestation of metabolic syndrome (9), and lipomatosis is well documented among patients with mitochondrial disorders (10). The present findings extend this literature by demonstrating these associations in a large, multicenter cohort.

### Biologic plausibility

**Figure 4:**
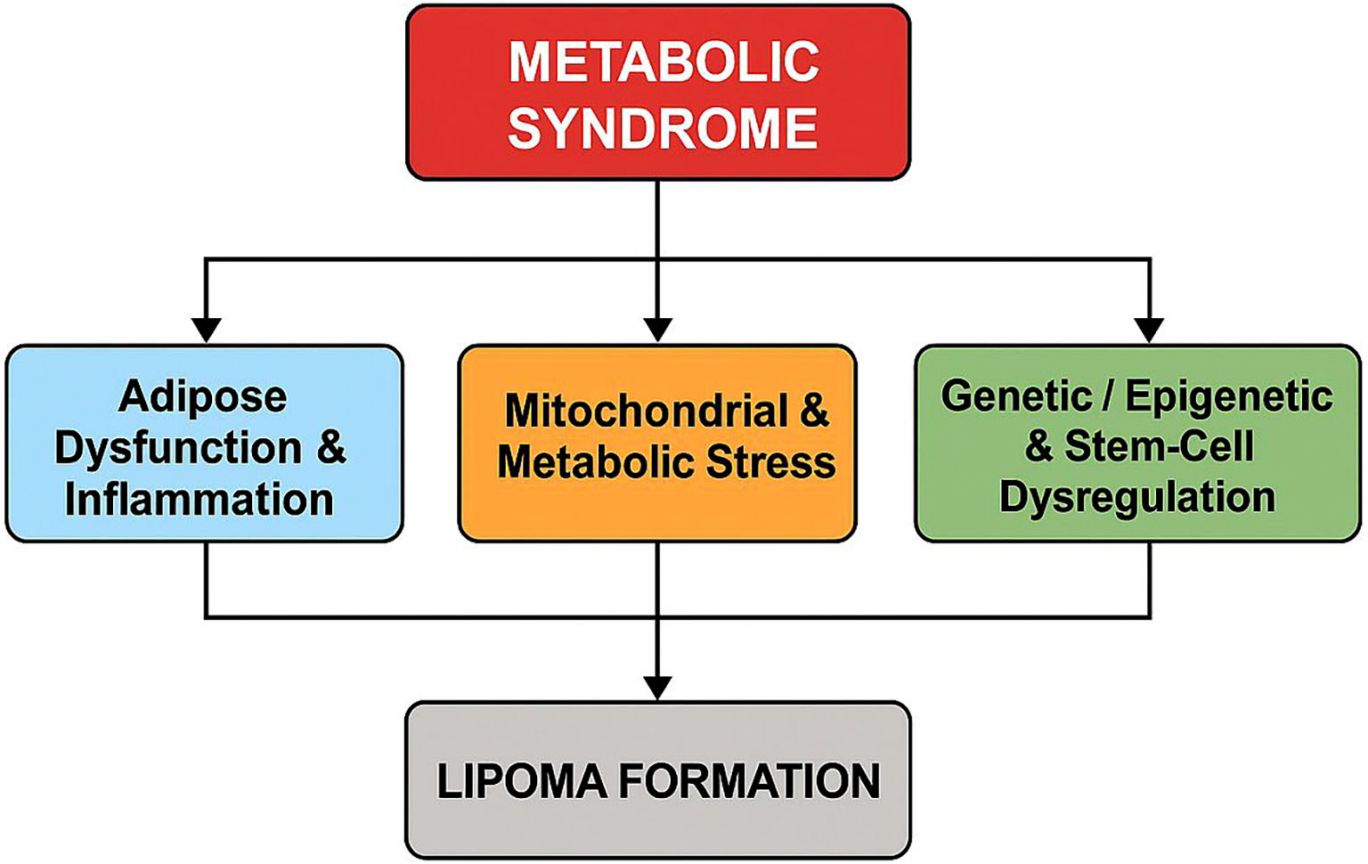
Putative pathways linking metabolic syndrome to lipoma formation. Conceptual model illustrating how metabolic syndrome may promote lipoma formation through three interacting axes: adipose dysfunction and inflammation, mitochondrial and metabolic stress, and genetic/epigenetic and stem-cell dysregulation.

Several mechanistic pathways could plausibly link metabolic syndrome (MetS) to lipoma development, although these were not evaluated in the present dataset (**Figure 4**). MetS is characterized by caloric excess, insulin resistance, and dyslipidemia, which promote adipocyte hypertrophy, local hypoxia, and HIF-1α–driven low-grade inflammation with TNF-α, IL-6 and MCP-1 signaling, engaging NF-κB/JAK–STAT pathways and worsening insulin resistance (11–13). Mitochondrial–immune crosstalk can amplify this loop (14), and chronically inflamed depots (e.g., spinal epidural lipomatosis) illustrate how reduced lipolysis and hyperinsulinemia may favor nodular fat expansion (9, 15). In parallel, nutrient overload and cytokine stress can impair β-oxidation/oxidative phosphorylation, generate ROS, and disrupt mitochondrial dynamics; MFN2 dysfunction, including the p.Arg707Trp variant, has been associated with symmetric lipomatosis, altered adipokines (elevated FGF-21, decreased leptin/adiponectin), and fragmented mitochondria (7, 16–21). MetS-related genetic/epigenetic and progenitor-cell changes, HMGA1/2 dysregulation, miR-27b activation of senescence pathways, and abnormalities in CD34^+^/PDGFRα^+^ adipogenic progenitors, may further bias adipose niches toward aberrant proliferation and differentiation (22–28). Collectively, these hypothesis-generating models provide biologic plausibility for our epidemiologic observation of stronger associations with metabolic clustering, but they require prospective and mechanistic studies for confirmation.

### Strengths and limitations

Strengths of our study include its large, multicenter cohort spanning three hospitals, use of harmonized electronic health records mapped to a common data model, and benchmarking against national survey data. These features enhance generalizability and reduce site-specific bias.

Several limitations warrant consideration. First, because this study is cross-sectional, temporality and causality cannot be inferred; shared metabolic or environmental factors may underlie both lipoma and metabolic traits. Second, the cohort was derived from Israeli hospitals, and patterns of metabolic syndrome and lipoma prevalence may differ across ethnic groups, lifestyles, and healthcare systems. Although the use of standardized diagnostic and laboratory criteria supports external validity, future studies should perform external validation in non-Israeli and multi-ethnic populations.

Third, we lacked an individual-level, lipoma-free control cohort and therefore relied on age- and sex-specific national benchmarks; future research should assemble hospital- or community-based control groups to enable covariate-adjusted modeling. Fourth, person-level covariates (e.g., BMI, medication use, lifestyle factors) were unavailable for the benchmark group, precluding multivariable regression and leaving residual confounding possible. Fifth, missing BMI and laboratory data reflected real-world testing patterns; we treated individuals with no diagnostic, medication, or measurement evidence for a given trait as out of scope rather than imputing missing values. This approach may have introduced selection bias, as patients undergoing laboratory testing may differ systematically from those without such data, with uncertain direction of effect. Finally, our hospital-based sample may overrepresent larger or symptomatic lipomas, and our trait definitions favored sensitivity over specificity, potentially introducing false positives.

### Clinical and translational implications

Given the graded excess we observed with metabolic clustering, a lipoma encounter should prompt targeted cardiometabolic screening, including blood pressure, BMI or waist circumference, lipid profile, and fasting glucose or HbA1c, particularly in adults ≥35 years or when multiple lesions or other metabolic features are present. Screening can be paired with brief lifestyle counseling and, when indicated, referral for risk-factor optimization.

Beyond epidemiology, hypothesis-generating data suggest that dietary fat composition and adipokine profiles modulate adipose inflammation and immune tone: experimental models report differential effects of saturated versus mono-/polyunsaturated fats, and adipokines such as adiponectin (versus IL-6 and TNF-α) have opposing inflammatory roles (29, 30). Although not assessed in our dataset, these observations motivate prospective and interventional studies to test whether metabolic optimization (including diet) can favorably modify the adipose microenvironment in patients with lipomas.

### Future directions

Because our analysis is cross-sectional, temporality and causality cannot be inferred. Future studies should therefore assemble an EHR-based lipoma-free matched-control cohort or a matched community cohort to enable covariate-adjusted modeling and clarify directionality. Prospective longitudinal designs with serial metabolic assessments could establish temporal ordering, while external validation in non-Israeli and multi-ethnic populations would assess generalizability. Mechanistic investigations integrating genomics, epigenomics, transcriptomics, and metabolomics of lipomas and adjacent adipose tissue are needed to test the proposed inflammatory, mitochondrial, and stem-cell pathways. Finally, interventional trials examining dietary fat composition and metabolic therapies may determine whether modifying the adipose microenvironment can reduce lipoma risk or improve metabolic health.

## Data Availability

The original contributions presented in the study are included in the article/**Supplementary Material**. Further inquiries can be directed to the corresponding author.
