# Supplementary material for: Lipomas are associated with a higher prevalence of metabolic syndrome components: a multicenter cross-sectional study
Source: Front Endocrinol (Lausanne). 2025 Dec 2;16:1721570. doi: 10.3389/fendo.2025.1721570 (PMC12705395; doi:10.3389/fendo.2025.1721570)
Supplement: Supplementary file 1 [file DataSheet1.docx]

**Appendix S1: Definitions of Metabolic Traits**

This appendix details the coding systems, laboratory cutoffs, and medication classes used to identify metabolic traits in the lipoma cohort. Unless otherwise stated, classification windows extended from 1 year prior to the lipoma index date through 3 months afterward.

**1. Dyslipidemia**

Diagnosis Codes (ICD-9-CM):

- Pure hypercholesterolemia: 272, 272.0, 272.00
- Pure hyperglyceridemia: 272.1
- Mixed hyperlipidemia: 272.2
- Hyperlipidemia: 272.4
- Unspecified disorder of lipoid metabolism: 272.9

Medications:
At least one prescription for a statin (HMG-CoA reductase inhibitor), e.g., simvastatin, atorvastatin, rosuvastatin, pravastatin, or equivalent.

Laboratory Measurements:

- Triglycerides ≥150 mg/dL (1.7 mmol/L)
- HDL cholesterol <35 mg/dL (0.9 mmol/L) in men or <39 mg/dL (1.0 mmol/L) in women

**2. Hypertension**

Diagnosis Codes (ICD-9-CM):

- Essential hypertension, malignant: 401, 401.0, 401.00
- Essential hypertension: 401.1
- Essential hypertension, benign: 401.9
- Secondary hypertension, renal origin, malignant: 405.0, 405.00, 405.01
- Secondary hypertension, renal origin, benign: 405.1, 405.11

Medications:
At least one prescription for a first-line antihypertensive:

- ACE inhibitors (e.g., ramipril, enalapril)
- ARBs (e.g., losartan, valsartan, candesartan)
- Calcium channel blockers (e.g., amlodipine, nifedipine)
- Thiazide diuretics (e.g., hydrochlorothiazide, chlorthalidone)

Note: β-blockers were excluded due to their multiple non-hypertensive indications.

Measurements:

- Systolic blood pressure ≥140 mmHg
- Diastolic blood pressure ≥90 mmHg

**3. Obesity**

Diagnosis Codes (ICD-9-CM):

- Obesity, unspecified: 278, 278.0, 278.00
- Morbid obesity: 278.01

Measurements:

- BMI ≥30 kg/m²
- When height and weight were available: BMI = weight (kg) / [height (m)]²

Note: Anti-obesity medications were not used as a criterion, since many are prescribed for type 2 diabetes rather than weight management.

**4. Type 2 Diabetes Mellitus (DM2)**

Diagnosis Codes (ICD-9-CM):

- 250.x0 or 250.x2
- Exclusions: type 1 diabetes codes, gestational diabetes (648.xx), and secondary diabetes (249.xx). General “diabetes mellitus” entries were included if no other type was specified.

Medications:
At least one prescription for a type 2 diabetes–specific agent (ATC code A10B), e.g., metformin, sulfonylureas, DPP-4 inhibitors, GLP-1 receptor agonists, SGLT2 inhibitors.

Laboratory Measurements:

- HbA1c ≥6.5% (valid without fasting)
- Fasting plasma glucose ≥126 mg/dL (7.0 mmol/L)
- OGTT (2-hour plasma glucose) ≥200 mg/dL (11.1 mmol/L)
- Random glucose ≥200 mg/dL (11.1 mmol/L) in the presence of classic symptoms (polyuria, polydipsia, weight loss)

**5. Rule-Based Classification Framework**

- Positive: Evidence from ≥1 domain (diagnosis, medication, abnormal lab).
- Negative: Normal laboratory values with no diagnosis or medication.
- Out of scope: Insufficient data (no diagnosis, no medication, no relevant lab); excluded from prevalence estimates.
